# Supplementary material for: Modeling glioblastoma heterogeneity as a dynamic network of cell states
Source: Mol Syst Biol. 2021 Sep 16;17(9):e10105. doi: 10.15252/msb.202010105 (PMC8444284; doi:10.15252/msb.202010105)
Supplement: Supplementary file 6 — Source Data for Figure 5 [file MSB-17-e10105-s004.zip › Figure5A_sourcedata/GSEA_3017/nosigdb_stateA.GseaPreranked.1622795026485/DOROSZKO_U3054MG_CLUSTER_4.html]

Details for gene set DOROSZKO\_U3054MG\_CLUSTER\_4[GSEA]

|  || Dataset | state53017 |
| Phenotype | NoPhenotypeAvailable |
| Upregulated in class | na\_pos |
| GeneSet | DOROSZKO\_U3054MG\_CLUSTER\_4 |
| Enrichment Score (ES) | 0.47062746 |
| Normalized Enrichment Score (NES) | 3.898723 |
| Nominal p-value | 0.0 |
| FDR q-value | 0.0 |
| FWER p-Value | 0.0 |
Table: GSEA Results Summary

  

Fig 1: Enrichment plot: DOROSZKO\_U3054MG\_CLUSTER\_4      
 Profile of the Running ES Score & Positions of GeneSet Members on the Rank Ordered List

  

| PROBE | GENE SYMBOL | GENE\_TITLE | RANK IN GENE LIST | RANK METRIC SCORE | RUNNING ES | CORE ENRICHMENT || 1 | CDK1 |  |  | 3 | 1.006 | 0.0137 | Yes |
| 2 | MKI67 |  |  | 4 | 0.926 | 0.0296 | Yes |
| 3 | KIF11 |  |  | 6 | 0.870 | 0.0434 | Yes |
| 4 | UBE2C |  |  | 7 | 0.793 | 0.0570 | Yes |
| 5 | MCM10 |  |  | 9 | 0.752 | 0.0688 | Yes |
| 6 | KIF20B |  |  | 10 | 0.749 | 0.0816 | Yes |
| 7 | CEP55 |  |  | 11 | 0.747 | 0.0945 | Yes |
| 8 | BUB3 |  |  | 12 | 0.740 | 0.1072 | Yes |
| 9 | MIF |  |  | 13 | 0.738 | 0.1199 | Yes |
| 10 | KPNA2 |  |  | 20 | 0.667 | 0.1242 | Yes |
| 11 | HIST1H4C |  |  | 21 | 0.659 | 0.1355 | Yes |
| 12 | ASPM |  |  | 27 | 0.639 | 0.1405 | Yes |
| 13 | PCLAF |  |  | 28 | 0.637 | 0.1515 | Yes |
| 14 | DNAJC9 |  |  | 34 | 0.615 | 0.1561 | Yes |
| 15 | CKS2 |  |  | 40 | 0.587 | 0.1602 | Yes |
| 16 | HMMR |  |  | 43 | 0.559 | 0.1675 | Yes |
| 17 | NCAPG |  |  | 44 | 0.556 | 0.1770 | Yes |
| 18 | HJURP |  |  | 47 | 0.539 | 0.1839 | Yes |
| 19 | CKS1B |  |  | 50 | 0.530 | 0.1906 | Yes |
| 20 | DBF4 |  |  | 55 | 0.514 | 0.1947 | Yes |
| 21 | SMC3 |  |  | 58 | 0.504 | 0.2010 | Yes |
| 22 | KIF14 |  |  | 59 | 0.503 | 0.2096 | Yes |
| 23 | MASTL |  |  | 60 | 0.499 | 0.2182 | Yes |
| 24 | MIS18BP1 |  |  | 64 | 0.495 | 0.2231 | Yes |
| 25 | CCNA2 |  |  | 65 | 0.495 | 0.2316 | Yes |
| 26 | TPX2 |  |  | 66 | 0.488 | 0.2400 | Yes |
| 27 | MYBL2 |  |  | 69 | 0.486 | 0.2460 | Yes |
| 28 | SGO2 |  |  | 73 | 0.478 | 0.2506 | Yes |
| 29 | HIST1H1B |  |  | 77 | 0.475 | 0.2552 | Yes |
| 30 | VRK1 |  |  | 82 | 0.470 | 0.2585 | Yes |
| 31 | UBE2T |  |  | 83 | 0.470 | 0.2666 | Yes |
| 32 | NUDT8 |  |  | 84 | 0.468 | 0.2747 | Yes |
| 33 | CENPF |  |  | 94 | 0.461 | 0.2719 | Yes |
| 34 | TOP2A |  |  | 95 | 0.457 | 0.2797 | Yes |
| 35 | HIST1H1D |  |  | 98 | 0.456 | 0.2852 | Yes |
| 36 | ZWINT |  |  | 106 | 0.447 | 0.2845 | Yes |
| 37 | AURKB |  |  | 108 | 0.446 | 0.2910 | Yes |
| 38 | CDCA8 |  |  | 115 | 0.437 | 0.2914 | Yes |
| 39 | KIF23 |  |  | 116 | 0.436 | 0.2989 | Yes |
| 40 | DEPDC1 |  |  | 119 | 0.432 | 0.3039 | Yes |
| 41 | MCMBP |  |  | 122 | 0.428 | 0.3089 | Yes |
| 42 | MELK |  |  | 123 | 0.427 | 0.3162 | Yes |
| 43 | JPT1 |  |  | 125 | 0.426 | 0.3223 | Yes |
| 44 | SGO1 |  |  | 126 | 0.425 | 0.3296 | Yes |
| 45 | BUB1B |  |  | 129 | 0.421 | 0.3345 | Yes |
| 46 | KNL1 |  |  | 133 | 0.418 | 0.3381 | Yes |
| 47 | HELLS |  |  | 135 | 0.417 | 0.3441 | Yes |
| 48 | RTKN2 |  |  | 141 | 0.413 | 0.3452 | Yes |
| 49 | PIMREG |  |  | 143 | 0.413 | 0.3511 | Yes |
| 50 | GTSE1 |  |  | 146 | 0.411 | 0.3558 | Yes |
| 51 | CCNB2 |  |  | 147 | 0.411 | 0.3629 | Yes |
| 52 | ANLN |  |  | 152 | 0.407 | 0.3651 | Yes |
| 53 | MAD2L1 |  |  | 153 | 0.406 | 0.3721 | Yes |
| 54 | KIFC1 |  |  | 160 | 0.402 | 0.3718 | Yes |
| 55 | ARHGAP11A |  |  | 162 | 0.399 | 0.3775 | Yes |
| 56 | KIF2C |  |  | 165 | 0.397 | 0.3820 | Yes |
| 57 | MCM7 |  |  | 171 | 0.394 | 0.3828 | Yes |
| 58 | NDC80 |  |  | 173 | 0.393 | 0.3884 | Yes |
| 59 | CKLF |  |  | 183 | 0.388 | 0.3843 | Yes |
| 60 | BUB1 |  |  | 188 | 0.384 | 0.3861 | Yes |
| 61 | CMSS1 |  |  | 191 | 0.381 | 0.3903 | Yes |
| 62 | KIF22 |  |  | 197 | 0.377 | 0.3908 | Yes |
| 63 | CDCA3 |  |  | 203 | 0.372 | 0.3913 | Yes |
| 64 | TRIP13 |  |  | 209 | 0.370 | 0.3917 | Yes |
| 65 | SMC4 |  |  | 217 | 0.367 | 0.3897 | Yes |
| 66 | SPC25 |  |  | 226 | 0.363 | 0.3864 | Yes |
| 67 | NCAPH |  |  | 232 | 0.359 | 0.3866 | Yes |
| 68 | PPIF |  |  | 248 | 0.350 | 0.3748 | Yes |
| 69 | DDIAS |  |  | 253 | 0.348 | 0.3760 | Yes |
| 70 | SDF2L1 |  |  | 257 | 0.345 | 0.3783 | Yes |
| 71 | ATIC |  |  | 258 | 0.344 | 0.3842 | Yes |
| 72 | FEN1 |  |  | 259 | 0.343 | 0.3901 | Yes |
| 73 | CENPJ |  |  | 270 | 0.339 | 0.3840 | Yes |
| 74 | LRR1 |  |  | 273 | 0.337 | 0.3875 | Yes |
| 75 | CLSPN |  |  | 274 | 0.336 | 0.3932 | Yes |
| 76 | ESCO2 |  |  | 282 | 0.333 | 0.3906 | Yes |
| 77 | ORC6 |  |  | 283 | 0.333 | 0.3963 | Yes |
| 78 | MTHFD2 |  |  | 287 | 0.330 | 0.3984 | Yes |
| 79 | CACYBP |  |  | 288 | 0.330 | 0.4041 | Yes |
| 80 | C21orf58 |  |  | 294 | 0.328 | 0.4038 | Yes |
| 81 | GGCT |  |  | 298 | 0.326 | 0.4058 | Yes |
| 82 | MND1 |  |  | 304 | 0.324 | 0.4054 | Yes |
| 83 | RAD18 |  |  | 307 | 0.324 | 0.4086 | Yes |
| 84 | BRCA2 |  |  | 308 | 0.323 | 0.4142 | Yes |
| 85 | CENPN |  |  | 310 | 0.323 | 0.4185 | Yes |
| 86 | FANCI |  |  | 319 | 0.319 | 0.4145 | Yes |
| 87 | TUBA1C |  |  | 322 | 0.318 | 0.4176 | Yes |
| 88 | TEDC1 |  |  | 324 | 0.317 | 0.4218 | Yes |
| 89 | RFC3 |  |  | 337 | 0.314 | 0.4129 | Yes |
| 90 | CDCA5 |  |  | 341 | 0.312 | 0.4147 | Yes |
| 91 | PRC1 |  |  | 350 | 0.310 | 0.4105 | Yes |
| 92 | POLR3K |  |  | 351 | 0.309 | 0.4159 | Yes |
| 93 | SLC20A1 |  |  | 361 | 0.306 | 0.4104 | Yes |
| 94 | ECT2 |  |  | 365 | 0.305 | 0.4121 | Yes |
| 95 | TTF2 |  |  | 367 | 0.305 | 0.4161 | Yes |
| 96 | FKBP5 |  |  | 368 | 0.304 | 0.4213 | Yes |
| 97 | BRCA1 |  |  | 373 | 0.302 | 0.4218 | Yes |
| 98 | CKAP2 |  |  | 379 | 0.301 | 0.4210 | Yes |
| 99 | RPA3 |  |  | 381 | 0.300 | 0.4250 | Yes |
| 100 | CSE1L |  |  | 382 | 0.300 | 0.4301 | Yes |
| 101 | RRM2 |  |  | 383 | 0.300 | 0.4353 | Yes |
| 102 | XRCC2 |  |  | 384 | 0.300 | 0.4404 | Yes |
| 103 | CENPW |  |  | 386 | 0.299 | 0.4444 | Yes |
| 104 | CDC6 |  |  | 391 | 0.297 | 0.4447 | Yes |
| 105 | PHF19 |  |  | 393 | 0.297 | 0.4486 | Yes |
| 106 | HMGA1 |  |  | 397 | 0.295 | 0.4501 | Yes |
| 107 | RACGAP1 |  |  | 399 | 0.293 | 0.4540 | Yes |
| 108 | HMGB2 |  |  | 424 | 0.288 | 0.4303 | Yes |
| 109 | POP7 |  |  | 427 | 0.287 | 0.4329 | Yes |
| 110 | NUSAP1 |  |  | 430 | 0.286 | 0.4354 | Yes |
| 111 | NUF2 |  |  | 434 | 0.285 | 0.4368 | Yes |
| 112 | MIS18A |  |  | 439 | 0.284 | 0.4369 | Yes |
| 113 | NCAPD2 |  |  | 440 | 0.283 | 0.4417 | Yes |
| 114 | LMNB1 |  |  | 442 | 0.282 | 0.4454 | Yes |
| 115 | CDC45 |  |  | 446 | 0.282 | 0.4467 | Yes |
| 116 | FBL |  |  | 451 | 0.281 | 0.4467 | Yes |
| 117 | GINS1 |  |  | 462 | 0.279 | 0.4396 | Yes |
| 118 | SMS |  |  | 463 | 0.278 | 0.4444 | Yes |
| 119 | MNS1 |  |  | 464 | 0.278 | 0.4492 | Yes |
| 120 | ATAD2 |  |  | 470 | 0.275 | 0.4480 | Yes |
| 121 | EZH2 |  |  | 471 | 0.275 | 0.4527 | Yes |
| 122 | MT2A |  |  | 473 | 0.275 | 0.4562 | Yes |
| 123 | SHCBP1 |  |  | 477 | 0.274 | 0.4573 | Yes |
| 124 | BLM |  |  | 490 | 0.272 | 0.4477 | Yes |
| 125 | PGP |  |  | 503 | 0.270 | 0.4381 | Yes |
| 126 | KIF4A |  |  | 504 | 0.269 | 0.4427 | Yes |
| 127 | UBE2S |  |  | 507 | 0.269 | 0.4449 | Yes |
| 128 | HAUS8 |  |  | 518 | 0.267 | 0.4376 | Yes |
| 129 | MANF |  |  | 521 | 0.266 | 0.4398 | Yes |
| 130 | CIP2A |  |  | 522 | 0.266 | 0.4444 | Yes |
| 131 | RCC1 |  |  | 528 | 0.265 | 0.4430 | Yes |
| 132 | SKA3 |  |  | 531 | 0.264 | 0.4452 | Yes |
| 133 | ALYREF |  |  | 535 | 0.264 | 0.4461 | Yes |
| 134 | KIF15 |  |  | 538 | 0.263 | 0.4482 | Yes |
| 135 | CDKN3 |  |  | 539 | 0.263 | 0.4528 | Yes |
| 136 | TTF1 |  |  | 541 | 0.262 | 0.4561 | Yes |
| 137 | SRSF2 |  |  | 542 | 0.262 | 0.4606 | Yes |
| 138 | STIL |  |  | 545 | 0.261 | 0.4627 | Yes |
| 139 | CENPO |  |  | 549 | 0.260 | 0.4636 | Yes |
| 140 | ERCC6L |  |  | 550 | 0.260 | 0.4680 | Yes |
| 141 | FBXO5 |  |  | 557 | 0.258 | 0.4653 | Yes |
| 142 | SPDL1 |  |  | 560 | 0.258 | 0.4674 | Yes |
| 143 | DDX39A |  |  | 562 | 0.258 | 0.4706 | Yes |
| 144 | PPP2R3B |  |  | 567 | 0.257 | 0.4703 | No |
| 145 | TMPO |  |  | 584 | 0.253 | 0.4556 | No |
| 146 | FAM111A |  |  | 618 | -0.259 | 0.4207 | No |
| 147 | NTM |  |  | 762 | -0.354 | 0.2566 | No |
| 148 | PEG10 |  |  | 917 | -0.587 | 0.0833 | No |
Table: GSEA details [plain text format]

  

Fig 2: DOROSZKO\_U3054MG\_CLUSTER\_4: Random ES distribution      
 Gene set null distribution of ES for **DOROSZKO\_U3054MG\_CLUSTER\_4**
